# A Simple Mathematical Model Based on the Cancer Stem Cell Hypothesis Suggests Kinetic Commonalities in Solid Tumor Growth

**DOI:** 10.1371/journal.pone.0026233

**Published:** 2012-02-17

**Authors:** Rodolfo Molina-Peña, Mario Moisés Álvarez

**Affiliations:** Centro de Biotecnología-FEMSA, Tecnológico de Monterrey at Monterrey, Monterrey, Nuevo León, México; University of Medicine and Dentistry of New Jersey, United States of America

## Abstract

**Background:**

The Cancer Stem Cell (CSC) hypothesis has gained credibility within the cancer research community. According to this hypothesis, a small subpopulation of cells within cancerous tissues exhibits stem-cell-like characteristics and is responsible for the maintenance and proliferation of cancer.

**Methodologies/Principal Findings:**

We present a simple compartmental pseudo-chemical mathematical model for tumor growth, based on the CSC hypothesis, and derived using a “chemical reaction” approach. We defined three cell subpopulations: CSCs, transit progenitor cells, and differentiated cells. Each event related to cell division, differentiation, or death is then modeled as a chemical reaction. The resulting set of ordinary differential equations was numerically integrated to describe the time evolution of each cell subpopulation and the overall tumor growth. The parameter space was explored to identify combinations of parameter values that produce biologically feasible and consistent scenarios.

**Conclusions/Significance:**

Certain kinetic relationships apparently must be satisfied to sustain solid tumor growth and to maintain an approximate constant fraction of CSCs in the tumor lower than 0.01 (as experimentally observed): (a) the rate of symmetrical and asymmetrical CSC renewal must be in the same order of magnitude; (b) the intrinsic rate of renewal and differentiation of progenitor cells must be half an order of magnitude higher than the corresponding intrinsic rates for cancer stem cells; (c) the rates of apoptosis of the CSC, transit amplifying progenitor (P) cells, and terminally differentiated (D) cells must be progressively higher by approximately one order of magnitude. Simulation results were consistent with reports that have suggested that encouraging CSC differentiation could be an effective therapeutic strategy for fighting cancer in addition to selective killing or inhibition of symmetric division of CSCs.

## Introduction

Fundamental and applied clinical research into cancer could greatly benefit from mathematical models that contribute to the basic understanding of this disease, to the planning of more efficient therapeutic strategies, or to the generation of accurate patient prognosis. This paper presents a general, simple, and flexible mathematical model, mechanistically based on the Cancer Stem Cell (CSC) hypothesis, that is capable of reproducing the dynamics observed during the exponential growth of a tumor.

Recently, the CSC hypothesis has gained credibility within the cancer research community [1]–[5]. In its simplest version, this hypothesis postulates that most tumors (if not all) arise by consecutive genetic changes in a small subpopulation of cells that have intrinsic characteristics similar to those of normal stem cells (SCs) [6]–[9]. A fast growing body of experimental evidence suggests that these so-called cancer stem cells (CSCs) are the drivers of cancer and are responsible for sustained tumor growth. Although no general consensus has yet been reached on several key aspects of the biology of CSCs, there is agreement in some of their distinctive features: (a) self-renewal capabilities, (b) potential for differentiation into the various cell subtypes of the original cancer, and (c) increased tumorigenesis [9]–[14].

Numerous researchers have reported the existence of CSC subpopulations in solid tumors [15]–[25]. CSCs have been reported to be more resistant to normal cancer therapies than are differentiated tumor cells (bulk tumor cells) [18], [19], [22], [25], [26]. Therefore, properly and selectively targeting CSCs could be one of the main lines of attack in a new wave of therapeutic strategies against cancer [5], [22], [27]–[29].

Although tumor growth has been a subject of intensive mathematical modeling in the last two decades, the concept of existence of a CSC population within tumors has been only recently included as an element in describing tumor growth [30]–[45]. Among these examples, different modeling approaches have been used, ranging from stochastic [35], [42], [45] to deterministic modeling [37], [41]. CSC-cancer modeling has frequently focused on the exploration of therapeutic strategies [36], [37], [41], [43]. For instance, Dingli and Michor [36] used mathematical modeling to demonstrate the importance of selective targeting of CSCs to improve the efficiency of cancer therapies. Similarly, Ganguly and Puri [39] formulated a model to evaluate chemotherapeutic drug efficacy in arresting tumor growth, based on the cancer stem cell hypothesis. Their results suggested that the best response to chemotherapy occurs when a drug targets abnormal stem cells. CSC based mathematical models have also been used to forecast the effect of specific therapeutic agents (and combinatory therapies). Several contributions have explored different aspects of the treatment with imatinib [37], [41], [43]. Mathematical modeling has also been used to gain understanding of fundamental issues underlying CSC biology [31], [32], [40], [42], [44], [45]. The biology of CSCs has not been fully elucidated and many questions still remain unresolved [16], [45]. In particular, some of these uncertainties are related to the dynamics of tumor growth. As an illustration, little is known about the balance between the multiple and complex cellular events that occur during the early stages of tumor progression. One of the central objectives of this work is to identify if some commonalities (or universal features) may exist with respect to the kinetics of early tumor growth. Experimentally studying the balance between the different cellular events involved on the process of tumor growth is not a trivial matter. Mechanistically based mathematical modeling might be highly useful for simulating the dynamics of cancer initiation and progression, the response to different therapies, and the evolution of resistance to drugs [30], as well as for gaining further fundamental understanding on the underlying dynamics of tumor growth.

In the present manuscript, we present a simple mathematical model that is designed to study the role of CSCs in tumor growth, with the aim of understanding the kinetic relationships between the different processes leading to exponential growth in solid tumors and evaluating possible therapeutic strategies for cancer treatment. We attempted to capture the key features of the known biological behavior of CSCs in a pseudo-chemical model, where cell division and death of the three cell subtypes considered are represented as “chemical reactions.” The intrinsic rates at which these reactions (cellular events) occur are the parameters of the model (*k_j_*) and are analogous to reaction rate kinetic constants. Based on an exploration of the parameter space of these kinetic constants, we derive conclusions related to their relative magnitudes. Some inferences regarding the fundamental biology of tumor growth and the effectiveness of some therapeutic strategies against cancer are discussed.

## Methods

### A pseudo-chemical model for tumor growth: underlying biological concepts

**Figure 1:**
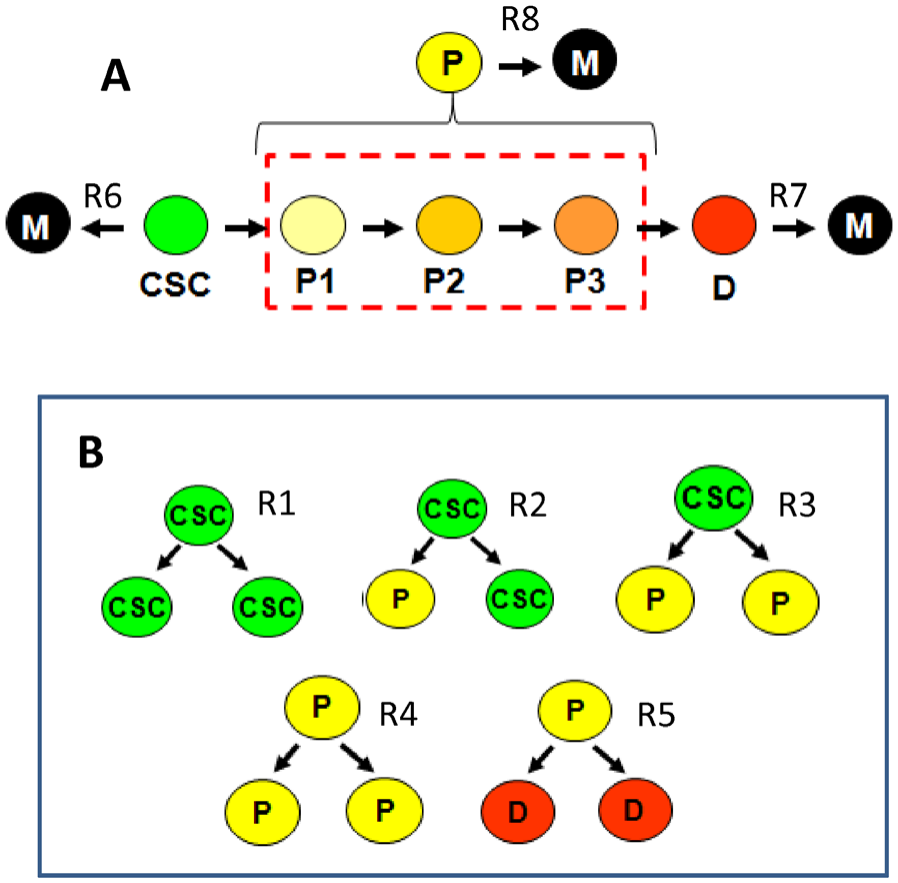
Basic assumptions of the model. (A) Different cell populations are found in solid tumors. For simplicity, the model considers only three cell compartments or differentiation stages (CSC = Cancer Stem Cells, P = progenitor cells, D = terminally differentiated cells, and M = dead cells). All the possible different stages of differentiation of progenitor cells (P1, P2, etc., have been lumped into the cell subtype P. CSC, P, and D cell subtypes undergo cell death through reactions R6, R7, and R8 respectively. (B) Cellular division events considered in the model: symmetrical self-renewal of cancer stem cells (R1); asymmetrical renewal of cancer stem cells (R2); symmetrical differentiation of cancer stem cells into progenitor cells (R3); symmetrical proliferation of progenitor cells (R4); and symmetrical differentiation of progenitor cells into fully differentiated cells (R5).

Tumors are a heterogeneous mix of cells, some of which exhibit SC-like characteristics [14], [16], [17], [34], [46]–[48]. It is probably more accurate to say that a tumor possesses a continuous spectrum of cell types, ranging from CSCs to more differentiated cells. In most of the previous modeling studies, the complexity of tumor tissue has been addressed by defining several cell subpopulations (typically from two to four), leading to compartment models [31], [32], [37], [40]. In order to reduce the complexity of the resulting model, only three subtypes of cells are considered: CSCs, transit amplifying progenitor cells (P), and terminally differentiated cells (D) (Fig. 1). This assumption is consistent with several experimental reports that simplify the cell heterogeneity found in cancer, in which three main cell subtypes are indentified [23], [48] with some variants in nomenclature; i.e., holoclones, meroclones, and paraclones [49]–[51].

In our model, events related to CSC self-renewal, to maturation of CSCs into P cells, to further differentiation to D cells, and to death of all cell subtypes, are represented as “chemical reactions” and are mediated by specific rate constants. These reactions occur in a system that has no nutrient limitations during the phase of exponential tumor growth. This assumption presumes that angiogenesis occurs at a rate that ensures the accessibility of nutrients sustain constant growth.

Framed in this way, the time evolution of all cellular subpopulations can be represented by a set of ordinary differential equations that have an analytical solution. In the following paragraphs, we introduce each of the cellular events considered for the construction of the model, and their representation in the form of “chemical reactions.”

Expansion of SCs can be accomplished through symmetric division [52], [53], whereby one CSC originates two CSCs:
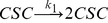
(*R*1)Alternatively, a CSC can undergo asymmetric division (whereby one CSC gives rise to another CSC and a more differentiated progenitor (P) cell). This P cell possesses intermediate properties between CSCs and differentiated (D) cells [2], [51], [54]–[56]:
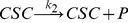
(*R*2)Both symmetrical and asymmetrical cell divisions of CSCs have been experimentally documented by staining of nuclear Oct-4 (a stem cell marker) [57]. Regulation of the ratio between symmetric and asymmetric division might possibly be crucial for the development and progression of cancer [44], [52].

CSCs may also differentiate to P cells by symmetric division [44], [58]:
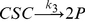
(*R*3)P cells can either self-renew, with a decreased capacity compared to CSCs, or they can differentiate to D cells [21], [31], [59], [60]:
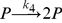
(*R*4)

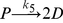
(*R*5)D cells do not have the capacity to proliferate [20], [51], [62], so their corresponding kinetic constant should be very small (here considered negligible). In addition, all cellular subtypes can undergo cell death:
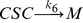
(*R*6)

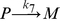
(*R*7)

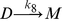
(*R*8)To establish the model, we followed a classical strategy used in chemical reaction engineering to describe a system of chemical reactions. For each cellular event, a “reaction rate” can be successively established: r_1_ = *k_1_*CSC; r_2_ = *k_2_*CSC; r_3_ = *k_3_*CSC; r_4_ = *k_4_*P; r_5_ = *k_5_*P; r_6_ = *k_6_*CSC; r_7_ = *k_7_*P; r_8_ = *k_8_*D.

Neglecting all terms related to transport of cells (to and from the tumor), the rate of accumulation of a cell subtype “*j*”, *dj/dt*, is equivalent to the net generation of that cell subtype, given by the addition of all reaction rates where that cell subtype is involved (i.e. produced or consumed).

For example, the cell species CSC is involved in the cellular events R1, R2, R3, and R6. In reaction R1, CSC is produced at a rate equivalent to 2r_1_, and consumed at a rate r_1_. In the cellular event R2, CSC is produced at a rate r_2_, and consumed at the very same rate r_2_. In the cellular event R3, CSC is consumed at a rate r_3_ due to differentiation into P. Similarly, in the cellular event R6, CSC is consumed at a rate r_6_ due to cell death.

The accumulation of CSCs within the system will be given by Eq. 1:
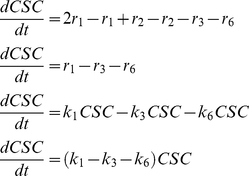
(1)Similarly, the accumulation of P and D cells within the system is expressed as:
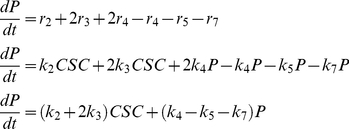
(2)And,
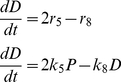
(3)This simple system of ordinary differential equations can be analytically solved to obtain the populations of each cell type (CSC, P, D) at any time, provided that a set of initial cell populations is specified (*CSCo*, *Po* and *D*).
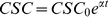
(4)

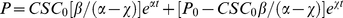
(5)

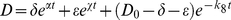
(6)Where
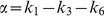
(7)

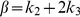
(8)

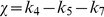
(9)

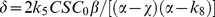
(10)

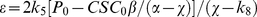
(11)The total number of tumor cells (*N*) will be the sum of the three cellular subtypes, assuming that dead cells are reabsorbed:
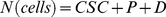
(12)The tumor volume can be calculated, considering that the effective volume contribution of a spherically shaped cell in a spherical tumor is 4.18×10^−6^ mm^3^/cell [62], as:
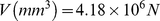
(13)This assumption implies that the tumor grows at constant cellular density, which might be reasonable during exponential tumor growth, when we have assumed that no nutrient transport limitations exist, and space constraints are not significant [63].

## Results and Discussion

### Significance of the parameters and constraints of the model

The model has one kinetic parameter per cellular event (or “reaction”). A brief discussion of the physical significance of these kinetic constants is pertinent here. In a typical elementary chemical reaction, the rate of appearance of a chemical species is proportional to the concentration of the reagents through a proportionality constant, the specific rate of reaction. Analogously, for our cellular system, the “rate of reaction” of each cellular event depends on both the number of precursor cells for that event and the proportionality constant *k_j_* that will multiply that number. For example, the rate of disappearance of CSCs, due exclusively to the occurrence of the cellular event R1, is r_1_, and is mediated by the kinetic parameter *k_1_*. Therefore, *k_1_* is an intrinsic reaction rate constant that indicates the natural predisposition of a cell, in this case a CSC, to divide symmetrically to originate two CSCs. In our model definition, *k_j_* is not equal to the growth rate of a cell subtype, but rather to the intrinsic proliferation rate associated with the frequency at which that particular cell subtype generally divides. To calculate the rate of accumulation of a particular cellular species (or net growth rate of that species), all terms where this species appears or disappears should be considered. For example, for the particular case of CSC, the associated rate of accumulation involves r_1_, r_3_, and r_6_ (see equation 1).

### Kinetic parameter grouping

The proposed kinetic model has eight independent variables. We simplified the analysis of the parameter space by grouping the eight kinetic constants of the model into three groups. Group I includes *k_1_*, *k_2_*, and *k_3_*; kinetic constants associated with cellular events that relate to CSC proliferation and differentiation. Group II includes *k_4_* and *k_5_*, since they mediate cellular events related to proliferation or differentiation of P cells. Finally, *k_6_*, *k_7_* and *k_8_* were included in Group III, as they describe cell death of each cell subtype.

In addition, some mathematical relationships among parameter groups and kinetic constants were defined. The value of k_1_, the rate constant for symmetric CSC renewal, was set equal to one. This is convenient, since all the rest of the *k_j_* values can now be defined (or scaled) relative to *k_1_*. In addition, it is helpful to define ratios between the kinetic parameters, namely Φ_2/1_, Φ_3/1_, Φ_4/1_, Φ_5/4_, Φ_6/1_, Φ_7/1_, and Φ_8/1_. For example, Φ_4/1_ is the ratio between *k_4_*/*k_1_*; biologically, it reflects the relative magnitude of the intrinsic kinetic constant governing symmetrical division of progenitor cells with respect to that related to symmetrical division of cancer stem cells. Similarly, Φ_5/4_ (*k_5_*/*k_4_*) indicates the relative magnitude between the rate constants associated to symmetrical division of progenitor cells to render two progenitor cells (R4) and symmetrical division of progenitor cells to produce two differentiated cells (R5). In this way, a vector of Φ_i/j_ values will define a complete set of *k_j_* values, and therefore a biological scenario for tumor growth. Illustratively, once *k_1_* is set to the unit value (*k_1_* = 1), the vector Φ_i/j_ = [Φ_2/1_, Φ_3/1_, Φ_4/1_, Φ_5/4_, Φ_6/1_, Φ_7/1_, Φ_8/1_] = [1.0, 0.01, 5.35, 0.8, 0.01, 0.1, 1.0] defines a scenario where *k_1_* = 1, *k_2_* = 1.0, *k_3_* = 0.01, *k_4_* = 5.35, *k_5_* = 4.28, *k_6_* = 0.01, *k_7_* = 0.1, *k_8_* = 1.0.

Although, *a priori*, all *k_j_* values are plausible, some constraints based on biological knowledge can be applied. For instance, in the present paper, the intrinsic apoptosis rate was considered to increase as cells become progressively more differentiated. Consequently, the greatest death rate corresponds to the most differentiated phenotype (D). On the other hand, CSCs have an extremely low apoptotic index [21], [53], [64]–[66]; therefore, the next constraints will be imposed for all simulations:
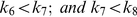
(14)In addition, experimentally, a minimum fraction of CSCs has been found to be maintained through the evolution of cancer [20], [61]. Normally, this fraction is lower than 1% of the total cell number [20], [25], [67]. Therefore, CSC/N<0.01 and d[CSC/N]/dt≈0 for all times. Finally, the fraction of P cells can be estimated from experiments in the literature to be approximately 0.2 [20], [68]. It is also well known that D cells constitute the majority of tumor cells [7], [20], [37]. Accordingly, we set the following expressions as constraints:
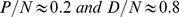
(15)The biology of CSCs has not been fully elucidated and many questions still remain unresolved [16], [45]. In particular, some of these uncertainties are related to the dynamics of tumor growth. As an illustration, little is known about the balance between the multiple and complex cellular events that occur during the early stages of tumor progression. One of the central objectives of this work is to identify if some commonalities (or universal features) may exist with respect to the kinetics of early tumor growth. Experimentally studying the balance between the different cellular events involved on the process of tumor growth is not a trivial matter.

The simple model that we proposed here allows for the study of the effect of variations in the relationships between the intrinsic kinetic values of each one of the cellular events defined (from R1 to R8). In our discussion, we place particular importance on the experimentally documented fact that the CSC fraction in a tumor is constant during tumor evolution, which is clear evidence of the crucial role of the CSC reservoir in tumor growth [20], [22], [25], [51]. Therefore, the constraint d[CSC/N]/dt≈0 becomes central to identifying biologically consistent and feasible solutions for the model.

### Feasible model solutions

In principle, one would expect that a vast number of combinations would render dynamical behaviors that would be consistent with experimental observations of the evolution of solid tumors. Based on our experience testing the model, the imposed constraints (namely d[CSC/N]/dt≈0; P/N≈0.2; *D*/N≈0.8; CSC/N<0.01) substantially limit the number of sets of parameter values that lead to feasible solutions.

**Figure 2:**
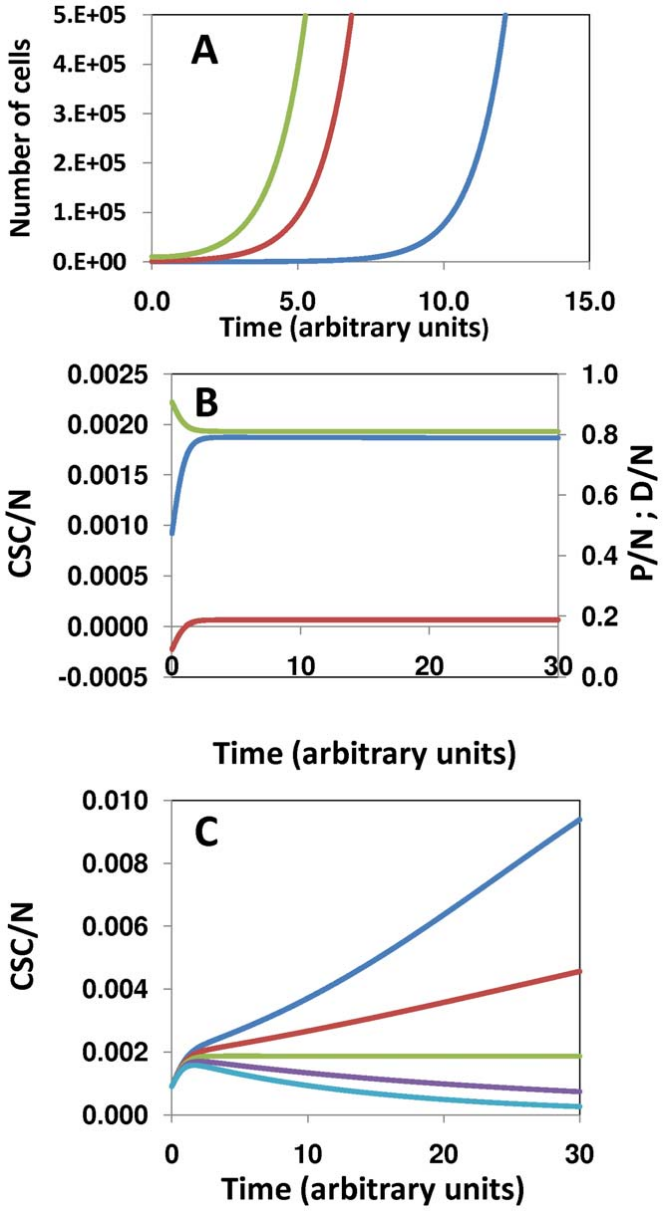
Model output. (A) The model estimates the time evolution of each one of the cell subpopulations considered, fully differentiated cells (D; blue line); progenitor cells (P; red line); and cancer stem cells (CSC; green line). (B) by plotting the cell fractions for each cell population (D/N; blue line), (P/N; red line), and (CSC/N; green line), it is possible to search for feasible and biologically consistent solutions (i.e. d[CSC/N]/dt = 0; C/N<0.01). (C) Only a relatively small set of parameter combinations result in solutions that satisfy the constraint d[CSC/N]/dt = 0. The solution defined by the vector Φ_i/j_ = [1.0, 0.01, 5.35, 0.8, 0.01, 0.1, 1.0] satisfy d[CSC/N]/dt = 0 only if when the specific value of Φ_4/1_ = 5.35 was used (green line). Values of Φ_4/1_ = *k_4_*/*k_1_*>3.5 (purple and light blue line) cause d[CSC/N]/dt<0; and values of Φ_4/1_ = *k_4_*/*k_1_*<3.5 (red and dark blue line) cause d[CSC/N]/dt>0.

As an illustration, let us consider the particular solution, obtained when the vector Φ_i/j_ = [Φ_2/1_,Φ_3/1_,Φ_4/1_,Φ_5/4_,Φ_6/1_,Φ_7/1_,Φ_8/1_] = [1.0, 0.01, 5.35, 0.8, 0.01, 0.1,1.0] is used. This scenario corresponds to one where *k_1_* = 1, *k_2_* = 1.0, *k_3_* = 0.01, *k_4_* = 5.35, *k_5_* = 4.28, *k_6_* = 0.01, *k_7_* = 0.1, *k_8_* = 1.0. The corresponding solution exhibits exponential growth, typically observed during the first stage of tumor growth (Figure 2A). After 30 arbitrary time units, all constraints are satisfied; namely, d[CSC/N]/dt≈0; P/N≈0.1875; *D*/N≈0.8106; CSC/N≈0.0018 (Figure 2B). Indeed, while exploring feasible solutions, we found the CSC/N *vs.* time plot to be particularly useful (see Figure 2C). For example, Figure 2C shows the dependence of the dynamics of CSC/N with respect to the value of Φ_4/1_. In this illustrative exercise, the rest of the Φ_i/j_ values remain constant, while Φ_4/1_ was progressively increased within the range of 5.0 to 5.7 units. Only specific value of Φ_4/1_ = 5.35 satisfied the condition of d[CSC/N]/dt = 0. Around the solution defined by the vector Φ_i/j_ = [1.0, 0.01, 5.35, 0.8, 0.01, 0.1, 1.0] other solutions exist that also satisfy d[CSC/N]/dt = 0; some of these have different CSC/N, P/N, and D/N steady state values. We found identification of these to be aided by trial and error investigations of the effect that small perturbations to this set of Φ_i/j_ values had on the quality of the solutions. Table 1 presents results from a series of simulation experiments where Φ_i/j_ values were varied around those that produced the solution previously discussed (Exp 0 in Table 1).

**Table 1:** Analysis of the effect of small perturbations around a particular solution.

Exp	Φ_1/1_	Φ_2/1_	Φ_3/1_	Φ_4/1_	Φ_5/4_	Φ_6/1_	Φ_7/1_	Φ_8/1_	d[CSC/N]/dt (*)	CSC/N @ss	P/N @ss	D/N @ss
0	1	1	0.01	5.35	0.8	0.01	0.1	1	**ss**	**0.0018**	0.1875	0.8106
1	1	1.5	0.01	5.35	0.8	0.01	0.1	1	(−)		0.1878	0.8105
2	1	0.5	0.01	5.35	0.8	0.01	0.1	1	(+)		0.1871	0.8107
3	1	1	0.015	5.35	0.8	0.01	0.1	1	(−)		0.1874	0.8108
4	1	1	0.005	5.35	0.8	0.01	0.1	1	(+)		0.1875	0.8103
5	1	1	0.01	5.45	0.8	0.01	0.1	1	(−)		0.186	0.8128
6	1	1	0.01	5.25	0.8	0.01	0.1	1	(+)		0.1891	0.807
7	1	1	0.01	5.35	0.85	0.01	0.1	1	**ss**	**0.0459**	0.1703	0.7836
8	1	1	0.01	5.35	0.75	0.01	0.1	1	**ss**	**0**	0.218	0.7819
9	1	1	0.01	5.35	0.8	0.1	0.1	1	(−)		0.1871	0.8127
10	1	1	0.01	5.35	0.8	0.001	0.1	1	(+)		0.1876	0.81
11	1	1	0.01	5.35	0.8	0.01	1	1	**ss**	**0.1435**	0.1608	0.6955
12	1	1	0.01	5.35	0.8	0.01	0.01	1	(−)		0.194	0.8057
13	1	1	0.01	5.35	0.8	0.01	0.1	10	**ss**	**0.0055**	0.5588	0.4356
14	1	1	0.01	5.35	0.8	0.01	0.1	0.1	**ss**	**0.0011**	0.1119	0.8869
15	1	1.5	0.005	5.35	0.8	0.01	0.1	1	**ss**	**0.0018**	0.1878	0.8102
16	1	0.8	0.012	5.35	0.8	0.01	0.1	1	**ss**	**0.0018**	0.1873	0.8107
17	1	1.5	0.01	5.325	0.8	0.01	0.1	1	**ss**	**0.0018**	0.1882	0.8098

(*) ss indicates that the solution reaches a steady state d[CSC/N]/dt = 0 in less than 30 arbitrary time units; (+) indicates that d[CSC/N]/dt>0 after 30 arbitrary time units; (−) indicates that d[CSC/N]/dt<0 after 30 arbitrary time units.

Rows corresponding to experiments 1 to 14 were built by varying only one Φ_i/j_ value at a time, while the rest were kept constant with respect to the reference case (Exp. 0). Column 10 indicates whether the CSC/N fraction reaches a steady state; that is, (d[CSC/N]/dt) = 0. If that is the case, the CSC/N fraction is indicated in column 11.

Some solutions, although satisfy d[CSC/N]/dt = 0, differ importantly in terms of their resulting cellular fractions at the steady state. For example, the solution of Exp. 7 (Table 1) is conducive to a steady state in which the cellular fractions CSC/N, P/N, and D/N are 0.0459, 0.1703, and 0.7836, respectively. In this particular case, the modification consisted of increasing the value of Φ_5/4_ (or *k_5_*/*k_4_*) from 0.80 to 0.85. This implies an increase of only 6.25% in the value of the intrinsic reaction rate constant of differentiation versus self-renewal of the subpopulation of progenitor cells. Not intuitively, the fraction of cancer stem cells increases as a result, due to the now higher mortality rate of differentiated cells induced by their higher cell numbers (r_8_ = *k_8_*[D]>r_7_ = *k_7_*[P]>r_6_ = *k_6_*[CSC]).

**Figure 3:**
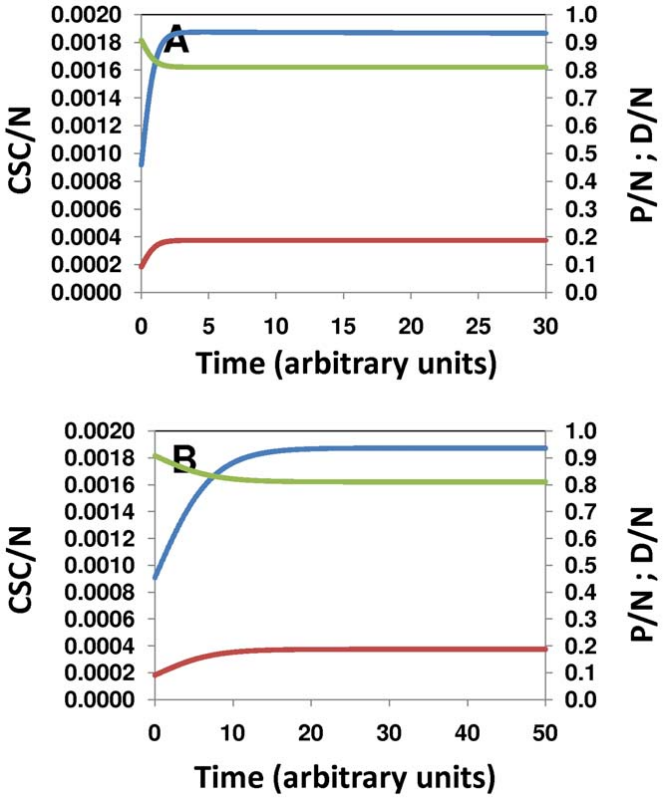
Feasible solutions for the model. Within the explored parameter space, once a feasible steady state solution is found, others can be found on the vicinity of a specific vector Φ_i/j_. (A) Solution for the vector Φ_i/j_ = [1.5, 0.005, 5.35, 0.8, 0.01, 0.1,1.0]. (B) Solution for the vector Φ_i/j_ = [1.5, 0.01, 5.325, 0.8, 0.01, 0.1, 1.0].

Other sets produce solutions with steady states similar to those of our reference case, previously mentioned (Φ_i/j_ = [1.0, 0.01, 5.35, 0.8, 0.01, 0.1, 1.0]; experiment 0 in Table 1). For example, the set Φ_i/j_ = [1.5, 0.005, 5.35, 0.8, 0.01, 0.1, 1.0] also produces a solution that satisfies d[CSC/N]/dt = 0, with a steady state characterized by the cellular fractions P/N≈0.1878; *D*/N≈0.8102; CSC/N≈0.0018 (Figure 3A, Exp. 15 in Table 1). We found several solutions (see Exp. 15, 16, and 17 in Table 1), with steady state values in the vicinity of P/N≈0.18, *D*/N≈0.81, and CSC/N≈0.0018, by modifying the value of two of the parameters Φ_i/j_ with respect to the values of the reference set (Exp. 0 in Table 1). To do so, we selected displacements (ΔΦ_i/j_) with opposite effects on the steady state P/N or CSC/N values (according to column No. 10 in Table 1). For example, in Exp. 15, an increase of 50% on the value of Φ_2/1_ was compensated by a proportional decrease (50%) on the value Φ_3/1_. This was an expected result: an increase in the rate of asymmetrical differentiation (r_2_) has to be balanced by a decrease in the rate of symmetrical differentiation (r_3_). Similarly, the opposite statement should hold. A decrease of 20% in the intrinsic rate of asymmetrical differentiation was compensated by a 20% increase in the rate of symmetrical differentiation (Exp. 16 in Table 1). Less intuitively, in Exp. 17 (see Figure 3B), an increment in the Φ_2/1_ value was balanced by a decrease on Φ_4/1_. In this case, a 50% increase in the rate of asymmetrical differentiation is equilibrated by a minor decrease (0.5%) in the intrinsic rate of both symmetrical proliferation (r_4_) and differentiation of progenitor cells (r_5_). Note that both the value of *k_4_* and *k_5_* are influenced by Φ_4/1_, since the value of Φ_5/4_ was left unmodified.

### Kinetic commonalities during exponential growth

While multiple sets of Φ_i/j_ could produce results consistent with the proposed set of constraints, those sets must comply with some general characteristics. For example, as illustrated before, the parameters Φ_2/1_ and Φ_3/1_ (and consequently *k_2_* and *k_3_*) are inversely related. To keep the overall fraction of cancer stem cells CSC/N constant over time, an increase on the value of *k_2_* must be balanced by a proportional decrease on *k_3_*, and *vice versa*.

This observation suggests a fine feedback biochemical control, and not necessarily a fixed ratio *k_2_*/*k_3_*. This result is relevant, since the experimental determination of the relative probability of occurrence of symmetric and asymmetric CSC division is difficult. The suggestion that regulation of the ratio between symmetric and asymmetric division may be crucial for the development and progression of cancer appears recurrently in the literature [33], [46], [52], [54], [55]. For example, Boman et al. [31], using a compartmental model, concluded that the only mechanism that can explain how CSC subpopulations can increase exponentially during colorectal cancer development involves an increase in symmetric SC cell division. This finding suggests that systemic therapies for effective treatment of cancers must act to control or eliminate symmetrical cancer SC division in tumors, while minimally affecting normal SC division in non-tumor tissues. In this respect, Turner et al. [45] have consistently concluded that symmetric division rates are the key in dictating brain tumor composition. Their results also suggested the importance of developing novel treatment strategies that specifically target the CSC subpopulation in brain tumors.

**Figure 4:**
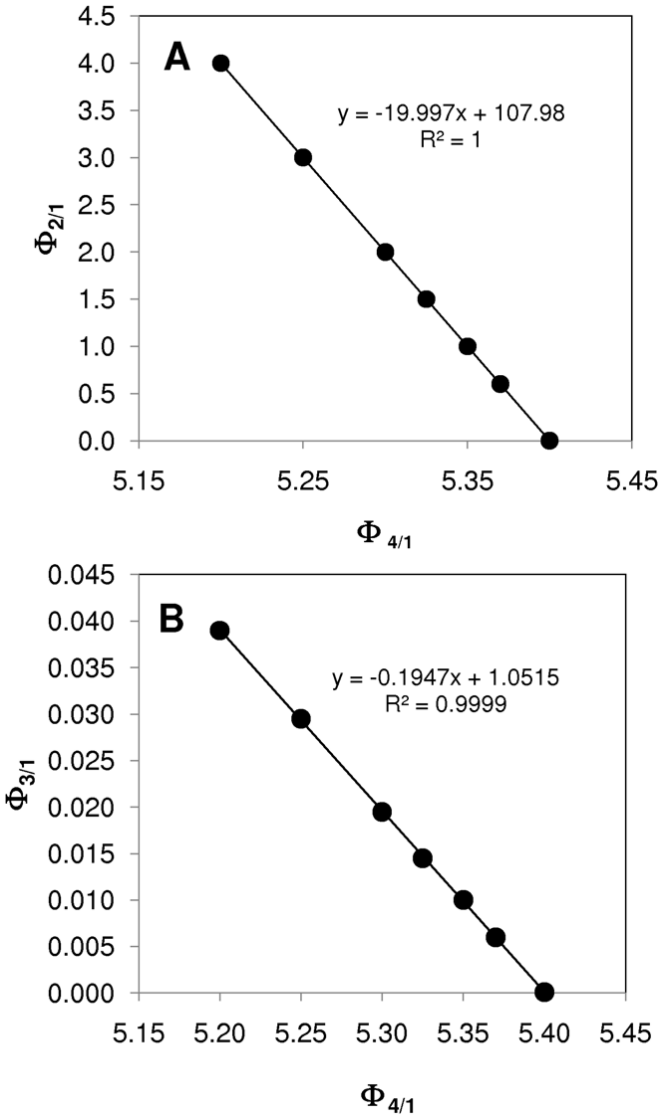
Linear relationships between model parameters. Certain linear relationships between CSC self-renewal and differentiation kinetic parameters allow identification of families of feasible solutions: (A) By increasing Φ_2/1_ while proportionally decreasing Φ_4/1_ a family of feasible model solutions can be found. Similarly, (B) Φ_3/1_ and Φ_4/1_ are linearly related.

Other kinetic relationships appear to be more stringent. Our results suggest that the ratio *k_4_*/*k_1_* has to be maintained in a very narrow band in order to sustain exponential tumor growth with constant CSC/N, P/N, and D/N fractions. For example, in our model, for the case where CSC/N≈0.0018, P/N≈0.18, and D/N≈0.81, the ratio *k_4_*/*k_1_* must be maintained at ≈5.35. Variations of less than 2.5% around this value must be compensated, for example, by important modifications to the value of *k_2_* (or more generally stated Φ_2/1_; see Figure 4A) or alternatively, *k_3_* (or more generally stated Φ_3/1_; Figure 4B).

We observed similar situations for steady states (d[CSC/N]/dt = 0) defined by different CSC/N, P/N, and D/N fractions. For example, the vector Φ_i/j_ = [1.5, 0.005, 3.6, 0.75, 0.01, 0.1, 1.0] produces a solution where d[CSC/N]/dt = 0, with CSC/N≈0.045, P/N≈0.25, and D/N≈0.70. Small variations in Φ_4/1_ = *k_4_*/*k_1_* = 3.6 disrupt the d[CSC/N]/dt = 0 condition, unless they are accompanied by an important adjustment to *k_2_*. Notice, however, that a 4.5% increase in CSC in a solid tumor is not consistent with previously reported experimental data. We could only find steady states (d[CSC/N]/dt = 0) with CSC/N<0.01, P/N≈0.20, and D/N≈0.80 when Φ_4/1_ = *k_4_*/*k_1_* was in the range of 5.18 to 5.40 (See Figure 4).

Indeed, we found a perfect linear correlation between the values of Φ_4/1_ and Φ_2/1_ that fulfill the conditions d[CSC/N]/dt = 0, CSC/N<0.0018, P/N≈0.20, and D/N≈0.80, namely Φ_2/1_ = −19.997Φ_4/1_+107.98 (Figure 4A). An analogous linear relationship exists between Φ_4/1_ and Φ_3/1_, namely Φ_3/1_ = −0.1947Φ_4/1_+1.0515 (Figure 4B). This suggests that the relative magnitude of the proliferation and differentiation rate constants for progenitor cells (*k_4_*+*k_5_*) must be at least half an order of magnitude above the analogous parameters for stem cells (CSC) in order to maintain exponential tumor growth while keeping (d[CSC/N]/dt = 0). Our results also suggest that the ratio between the intrinsic constant rate for symmetric proliferation of progenitor cells and the analogous parameter for stem cells symmetric self-renewal (Φ_4/1_ = *k_4_*/*k_1_*) should be approximately half an order of magnitude (between 5.10 and 5.4), independently of the values of *k_2_* and *k_3_*. We also observe that, to maintain tumor growth, the constant for symmetric cancer stem cell renewal should be in the same order of magnitude as the sum of *k_2_* and *k_3_*.

The time evolution of the rates of proliferation of CSCs, progenitors, and terminally differentiated cells (*dCSC/dt*, *dP/dt*, and *dD/dt* respectively) can be calculated using equations (1), (2), and (3). Despite the fact that the *k_j_* values associated with CSC events are of the order of magnitude, and in some cases even higher, than *k_j_* values corresponding to P and D cells, the number of stem-like cells is much smaller than numbers of progenitors or differentiated cells. Consequently, the product (*k_1_CSC*) and the global rate of proliferation *(dCSC/dt)* are one or two orders of magnitude smaller than those rates for more differentiated cells during all stages of the exponential growth of a tumor. In addition, early in the development of a tumor, the ratios between the global growth rates of the different cell subtypes achieve equilibrium. According to our simulation results, (*dP/dt*)/(*dCSC/dt*)≈100 and (*dD/dt*)/(*dP/dt*)≈4. Therefore, (*dD/dt*)/(*dCSC/dt*)≈400. This equilibrium of growth rates between cell subtypes must be achieved if balanced tumor growth is to maintain constant ratios of cell subpopulations. Again, these ratios between growth rates of different cell subpopulations are not easy to calculate *in vivo*.

### The role of cell death

**Figure 5:**
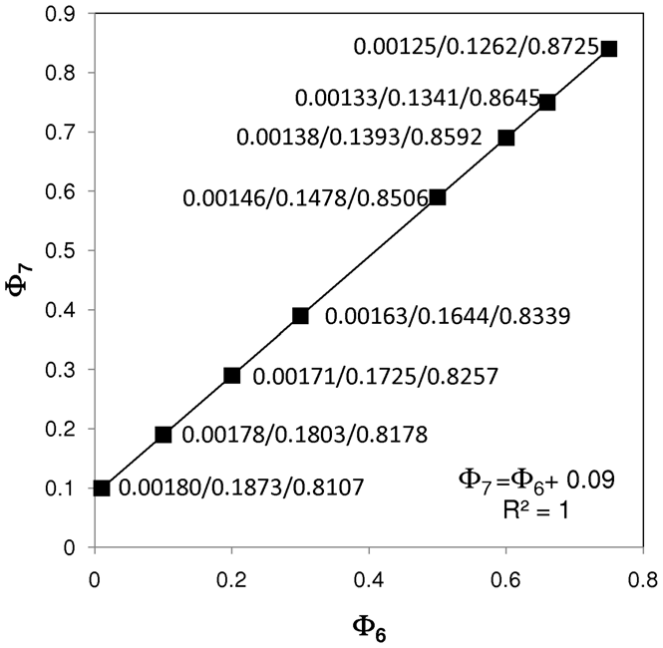
Linear relationships between model parameters. The intrinsic apoptotic rates of CSC and P cells are also linearly related. Proportional increases in Φ_6/1_ and Φ_7/1_ can reveal a family of feasible model solutions.

The model is sensitive to the ratios between cancer stem cell self-renewal and cell death (Φ_6/1_, Φ_7/1_, Φ_8/1_). The initial assumption of *k_6_*<*k_7_*<*k_8_* should be satisfied in order to observe constant cell fractions throughout exponential growth. This is consistent with several experimental reports. For instance, it has been observed that the apparent rate of death in prostate CSC is much lower than in other tumor cell subtypes [20], [51]. We also found that the Φ_6/1_, Φ_7/1_, Φ_8/1_ values should be in the range of 0.01 to the same order of magnitude as *k_1_* (Φ_8/1_≈1) to produce solutions where d[CSC/N]/dt = 0 and CSC/N<0.01. Provided that these two criteria are satisfied (*k_6_*<*k_7_*<*k_8_* and Φ_8/1_≈1), families of solutions satisfying d[CSC/N]/dt = 0 and CSC/N<0.01, but with different P/N and D/N values, can be obtained. For example, Figure 5 presents one of these solution families, where *k_1_* = 1 and vector Φ_i/j_ was initially set as Φ_i/j_ = [080, 0.012, 5.35, 0.8, 0.01, 0.1, 1.0] to locate a first condition satisfying d[CSC/N]/dt = 0 and CSC/N<0.01. Other points of the same series can be found by trial and error, by progressive (and proportional) changes in the Φ_6/1_ and Φ_7/1_ values while keeping the rest of the parameters constant. Specifically, we identified an entire family of solutions that obeys the linear relationship Φ_7_ = Φ_6_+0.09. As the Φ_6/1_ and Φ_7/1_ values increase, the CSC fraction decreases from 0.0018 to 0.00125, and the fraction of differentiated cells increases from 0.8107 to 0.8725.

### Model fitness to experimental data sets

**Figure 6:**
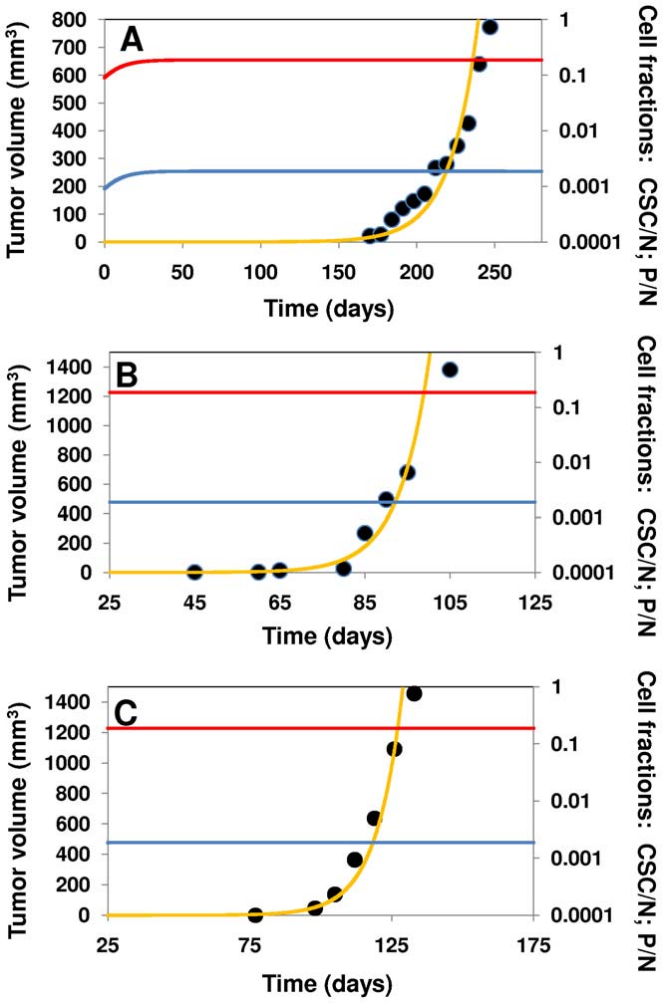
Three experimental data sets, corresponding to the different scenarios referred to in the text, were used to validate the model. A comparison between the experimental data (• black circles) and the model curve-fit (yellow solid line) is provided for each set. The CSC/N fraction (blue line) and P/N fraction (red line) are plotted for each experimental scenario. For each simulation, the vector Φ_i/j_ = [Φ_2/1_, Φ_3/1_, Φ_4/1_, Φ_5/4_, Φ_6/1_, Φ_7/1_, Φ_8/1_] = [1.0, 0.01, 5.35, 0.8, 0.01, 0.1, 1.0] was multiplied by a different scaling factor (17.5×Φ_i/j_; 5.0×Φ_i/j_; and 7.0×Φ_i/j_ respectively). (A) A human prostate tumor transplanted into a mouse model [69]; it is assumed that the original percentage of CSCs was 1%; (B) 2000 CSCs from a breast primary tumor implanted in NOD/SCID mice [25]; and (C) 1×10^5^ colon CSCs isolated and implanted in NOD/SCID mice [13]. For all simulations, the vector Φ_i/j_ = Φ_i/j_ = [Φ_2/1_, Φ_3/1_, Φ_4/1_, Φ_5/4_, Φ_6/1_, Φ_7/1_, Φ_8/1_] = [1.0, 0.01, 5.35, 0.8, 0.01, 0.1, 1.0] was used.

The model is flexible enough to allow proper adjustment to a wide range of possible tumor growth scenarios. Here, to validate the model, we selected three different tumor growth experimental data sets available from previous literature [13], [25], [69]. In all cases, the same Φ_i/j_ vector was used to describe the ratios between the kinetic parameters of the model, namely Φ_i/j_ = [Φ_2/1_, Φ_3/1_, Φ_4/1_, Φ_5/4_, Φ_6/1_, Φ_7/1_, Φ_8/1_] = [1.0, 0.01, 5.35, 0.8, 0.01, 0.1, 1.0]. After proper scaling of the y axes (by multiplying by a different scaling factor in each case), the model reproduces the three experimental data sets with an R-square greater than 0.97 (Figure 6). This suggests that, although the values for the intrinsic kinetic rates of each tumor might be different, the relationships between them (all Φ_i/j_ values) could be approximately common among different cancer types. For all simulations, all constraints were satisfied for each set of experimental data, particularly the condition of a constant CSC cell fraction lower than 1% (specifically 0.0018).

We should emphasize that our model is capable of reproducing the evolution of tumor growth only during its exponential phase. For the experimental sets that we had analyzed, this means up to a size of 1500 mm^3^; clinically, a medium size solid tumor. Tumors of this size quite often contain necrotic tissue at their central core. Our model does not distinguish between living and dead tissue, and only provides an overall volume based on a very naive and simple approximation of equal spherical volume contribution of each cell (dead or alive) within the tumor. Although simple and unrealistic, this assumption has yielded a good agreement with experimental sets for our descriptive purposes.

### Contrasting strategies to combat cancer

Most of the currently used chemotherapy and radiotherapy strategies against cancer are unable to distinguish between different tumor cell types, or even healthy and tumor cells, killing them all unselectively. Some reports also indicate that CSCs are particularly resistant to conventional therapeutic procedures [18], [19], [23], [26], [51]. Friel et al. [19] reported that CSCs isolated from human EnCa were particularly resistant to Paclitaxel, a widely used chemotherapeutic anticancer agent. Kang et al. [18] found that CSCs from GBM were radio-resistant when exposed to radiation dosages that killed other tumor cell subpopulations. Eyler and Rich [26] and Dylla et al. [23] reported additional evidence of CSC resistance to conventional therapies. More recently, Tan et al. demonstrated that holoclone forming cells from pancreatic tumors (with stem cell characteristics) exhibit much higher chemoresistance to gemcitabine and 5-FU than meroclones and paraclones [51].

This enhanced resistance of CSCs would explain the observed aggressive regeneration of tumors after treatment. The remaining CSC population would grow exponentially (having an abundant amount of nutrients) and would readily regenerate the other subpopulations that form the bulk of the tumor.

**Figure 7:**
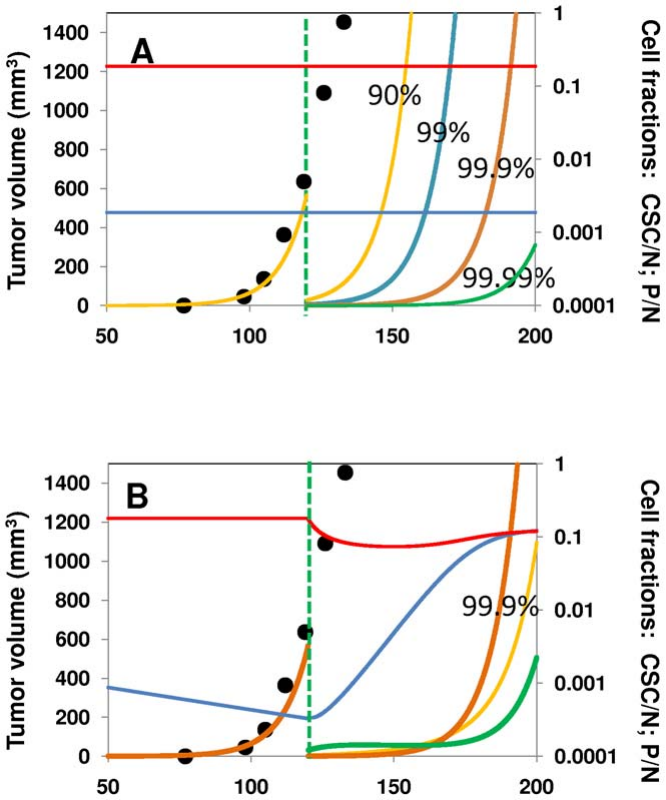
Different strategies against tumor progression are explored using the proposed model. (A) The progression of a tumor without intervention [15] is depicted from day 0 to day 120 (black circles and yellow curve). Different percentages of all tumor cells (CSC, P and D) are unselectively eradicated at day 120: 90% (yellow line); 99% (blue line); 99.9% (orange line) and 99.99% (green line). The CSC/N fraction (blue line) and P/N fraction (red line) remain unaltered for all unselective treatments. (B) Effective alternative therapeutic strategies to treat cancer might include enhancement of differentiation over CSC self-renewal: i.e., 10% increase in Φ_5/4_ (yellow line) or 15% increase in Φ_4/1_ (green line). The CSC/N fraction (blue line) and P/N fraction (red line) are plotted for the case of a 15% increase. The case where 99.9% of all tumor cells are eradicated unselectively at day 120 is included as a reference (orange line).

Figure 7A shows the behavior of a solid tumor when treated with a non-specific therapy such that 90%, 99%, 99.9% or 99.99% of the solid tumor cells are eradicated at a particular time, let us say 120 days. For illustrative purposes, the base case we have chosen is the case illustrated in Figure 6C [13]. In all instances, after an incubation time, the tumor relapses to exhibit practically the same progression profile originally followed. Even in the case of the most effective treatment, which kills 99.99% of all tumor cells (including 99.99% of CSCs), the tumor is able to reinitiate growth in a relatively short time (within 50 days).

An intuitive therapy to eradicate tumors, widely suggested in recent literature [22], [23], [24], [26]–[29], [31], [46], [70]–[72], relates to the targeting of CSCs, killing them selectively through treatment (i.e., augmenting *k_6_* significantly over *k_7_*, and *k_8_*) or, alternatively, preferentially killing stem cells at a particular time. Other authors have evaluated this strategy using mathematical models; for example, Dingli and Michor [36] designed a simple mathematical model to demonstrate the importance of eliminating tumor stem cells. The authors explored different therapeutic scenarios to illustrate the properties required in novel anti-cancer agents for successful tumor treatment. Their results indicated that successful therapy must eradicate tumor stem cells. Similarly, Ganguly and Puri [39] formulated a mathematical model to evaluate chemotherapeutic drug efficacy in arresting tumor growth based on the cancer stem cell hypothesis. Their results also suggest that the best response to chemotherapy occurs when a drug targets abnormal stem cells.

However, our simulations show that this therapeutic avenue, although superior to unselective treatments, is only effective if progenitor cells (P) are also targeted. This is, for the time window that our model is applicable, the exponential tumor growth phase, both cancer stem cells and progenitor cells have to be eradicated to stop tumor growth. This can be easily seen by examining equation 2 [*dP/dt = (k_2_+2k_3_)CSC+(k_4_−k_5_−k_7_)P* ]. In that equation, the rate of accumulation of the subpopulation of progenitor cells depends on two terms, one affected by the number of CSCs and the other dependent on the number of P cells. Therefore, even in the complete absence of CSCs, the term depending on P has to be negative for P to decrease. That term, namely *(k_4_−k_5_−k_7_)P*, can only be negative if *k_4_ <k_5_+k_7_*. For the set of Φ_j/i_ values that we have chosen (based on the rational that we had explained before: d[CSC/N]/dt = 0 with CSC/N<0.01) this condition is not met. The need to not only specifically target CSC, but also the progenitor subpopulation to effectively detain tumor progression has been suggested before in literature [73], also at the light of a mathematical argument.

Conceivably, in the long term, selective killing of all cancer stem cells will be sufficient to eradicate the tumor, since CSCs are the ultimate cell reservoir responsible for sustained growth. At the end of exponential growth phase, the relative weight of *k*_7_ has to increase (or alternatively the ratio Φ_5/4_ = *k_5_*/*k_4_* will change) due to causes such as oxygen mass transfer or nutrient limitations. We plan to include this functionality in a later version of our model.

This last comment on the relative ratio Φ_5/4_ = *k_5_*/*k_4_* is useful to introduce a less intuitive therapeutic strategy. Promotion of differentiation has been suggested for clinical practice [74], [75]. Using the proposed model, we explored variants of this approach. Indeed, augmenting Φ_5/4_ = *k_5_*/*k_4_* appears to be therapeutically promising. In biological terms, this means increasing the relative intrinsic rate of progenitor cell differentiation with respect to the rate of progenitor cell renewal. Interestingly, a very modest 10% increase in Φ_5/4_ (from 0.8 to 0.9), while keeping the rest of the Φ_i/j_ values fixed, retards tumor relapse more effectively than does the unselective tumor treatment (see Figure 7B). Similarly, increasing the ratio Φ_4/1_ = *k_4_*/*k_1_* and promoting a tumor richer in P cells also is another effective therapeutic strategy. Indeed, the delay induced by an increase of 15% on Φ_4/1_ = *k_4_*/*k_1_* is comparable to that caused by unselective eradication of 99.99% of the tumor cell mass (Figure 7B).

### Concluding remarks

In summary, in this study, we presented a first version of a conceptually simple model, with an analytical solution, that is capable of describing the basic kinetic features of tumor growth during the exponential phase and that is consistent with the Cancer Stem Cell hypothesis. Three cell subpopulations were considered: CSCs, progenitors (P), and terminally differentiated (D) cells. Each event related to cell division or death of each one of these subpopulations has been represented and modeled as a chemical reaction. This resulted in an analytically solvable set of ordinary differential equations that describes the time evolution of each cell subpopulation, as well as the overall tumor volume evolution during exponential growth.

Although, in principle, an infinite set of combinations of model parameter can be studied, we found that only a limited set of model solutions is feasible if some biologically sound constraints are imposed. For example, if we accept that the fraction of cancer stem cells during the exponential phase of the tumor is practically constant and no greater than 0.01 (namely d[CNC/N]/dt = 0 with CSC/N<0.01), then only a reduced set of solutions is feasible. The analysis of those sets suggests kinetic commonalities in solid tumor growth: (a) the rates of symmetrical and asymmetrical CSC renewal must be in the same order of magnitude; (b) the intrinsic rate of renewal and differentiation of progenitor cells should be half an order of magnitude higher than the corresponding intrinsic rates for cancer stem cells; (c) the rates of apoptosis of the CSC, P, and D cells are progressively higher by approximately one order of magnitude. The flexibility of the model was tested by fitting experimental data sets from three different tumor growth scenarios. After adequate scaling, a single set of kinetic parameters can be used for adequate reproduction of different tumor growth cases.

We do not claim that our model renders accurate information about the kinetics of solid tumor formation, but we observe that it does provide insight into several underlying kinetic behaviors of solid tumor growth that would be difficult to directly study experimentally. As illustrated in this work, the model can even be useful for anticipating the effect of different therapeutic strategies (available or potential) against cancer.
